# Operationalizing the One Health approach in a conflict-affected setting: A scientometric review of policy foundations, systemic gaps, and future pathways in Ukraine

**DOI:** 10.14202/vetworld.2026.389-408

**Published:** 2026-01-31

**Authors:** Anton Gerilovych, Nadiia Shevchenko, Oleksandr Pishchanskyi, Halyna Aliekseieva, Mykhailo Rosada, Iryna Gerilovych, Oksana Okaievych

**Affiliations:** 1 Institute for Problems of Cryobiology and Cryomedicine of the NAS of Ukraine, Kharkiv, Ukraine; 2 One Health Scientific and Research Institute, Kharkiv, Ukraine; 3 State Scientific and Research Institute for Laboratory Diagnostics and Veterinary and Sanitary Expertise, Kyiv, Ukraine; 4 One Health Institute, NGO, Kharkiv, Ukraine; 5 National Scientific Center “Institute for Experimental and Clinical Veterinary Medicine”, Kharkiv, Ukraine

**Keywords:** antimicrobial resistance, conflict and health, environmental health, One Health, public health security, Ukraine, veterinary public health, zoonotic diseases

## Abstract

**Background and Aim:**

The One Health approach integrates human, animal, plant, and environmental health through multisectoral collaboration and is increasingly recognized as essential for addressing zoonotic diseases, antimicrobial resistance (AMR), food security, and ecosystem degradation. Ukraine has formally adopted One Health principles through national strategies and international partnerships; however, the ongoing full-scale military conflict has profoundly disrupted health, veterinary, and environmental systems, challenging effective implementation. This study aimed to evaluate the current status, achievements, and constraints of the One Health approach in Ukraine, with particular emphasis on the effects of armed conflict on governance, surveillance capacity, and intersectoral coordination, and to outline strategic priorities for strengthening One Health resilience.

**Materials and Methods:**

A mixed-methods approach was used, combining bibliometric analysis of Scopus-indexed literature on zoonoses, AMR, food security, and environmental safety with targeted case studies and a review of policy documents. National legal frameworks, international guidelines, and reports from global organizations were systematically analyzed to assess institutional capacity and operational readiness.

**Results:**

Ukraine has established a solid policy foundation for One Health, notably through the national Strategy for Biosafety and Biosecurity, which is grounded in the One Health principle and aligned with quadripartite frameworks. Active initiatives address priority zoonoses (rabies, leptospirosis, tuberculosis), AMR surveillance, and food safety. Nevertheless, implementation remains fragmented. Armed conflict has caused extensive damage to laboratories, displaced the workforce, created surveillance blind spots, and disrupted multisectoral communication. AMR trends have intensified due to healthcare strain, while environmental and plant health components remain under-integrated despite their relevance to food security and long-term resilience. The Ukrainian experience demonstrates that policy commitment alone is insufficient in the context of conflict. Effective One Health operationalization requires institutionalized governance mechanisms, interoperable surveillance systems, and sustained investment in human resources and laboratory infrastructure. Environmental and plant health integration remains a critical gap.

**Conclusion:**

Reinforcing the One Health framework is essential for Ukraine’s recovery and long-term health security. Sustained international technical and financial support, coupled with national institutionalization of One Health principles, is crucial to rebuilding integrated surveillance, mitigating biological risks, and enhancing resilience in conflict-affected settings.

## INTRODUCTION

Over recent decades, research in human and animal health has faced increasingly complex challenges driven by profound global environmental and geopolitical changes. These emerging pressures often surpass the cumulative impact of more traditional health concerns. Many of these challenges are closely associated with population growth and its downstream effects, including rapid urbanization, large-scale migration, intensification of agricultural production, ecosystem disruption, and the globalization of trade and transport networks. In parallel, recent years have seen unprecedented increases in global food and energy prices, creating particularly severe conditions for developing countries, especially in the context of armed conflicts such as the ongoing aggression of the Russian Federation against Ukraine [1, 2].

In addition to these socioeconomic disruptions, scientists have warned of impending crises linked to the unsustainable use of natural resources, particularly freshwater availability and pollution, which may become drivers of instability and even armed confrontation [3]. Global climate change, the emergence and re-emergence of infectious diseases, and the accelerating spread of antimicrobial resistance (AMR) further underscore that human, animal, and environmental health cannot be effectively addressed in isolation [4, 5]. These rapidly evolving threats, together with the inherent interconnectedness among humans, domestic and farm animals, wildlife, and ecosystems, emphasize the urgent need for comprehensive and integrative approaches to health and environmental protection.

Approximately 10–15 years ago, the One Health concept expanded beyond its original focus on human and animal health to explicitly include plant health, recognizing the central role of crop production and ecosystem stability in ensuring food security and global well-being [6]. More recently, there have been growing calls to further broaden the One Health framework to reflect the complex interconnections among agriculture, plant and animal health, food safety, environmental sustainability, and public health [7–9].

Within this global context, Ukraine has begun integrating the One Health approach into national strategies, research agendas, and educational programs, positioning it as a key framework for addressing biosecurity threats, zoonotic diseases, and ecological resilience amid both natural challenges and war-associated pressures, while fulfilling international commitments.

This review is significant as it provides the first comprehensive, evidence-based evaluation of the One Health approach in the Ukrainian context, integrating human, animal, and plant health together with ecosystem well-being into a unified analytical framework. Current conditions in Ukraine, including ongoing armed conflict, disruption of public health and veterinary infrastructure, increasing burdens of zoonotic diseases, rising AMR [10–14], and environmental degradation [15], highlight the urgent need for coordinated multisectoral action.

By synthesizing international standards [12, 13, 16], national legislation [17–19], scientific programs, and practical case studies, this review identifies critical gaps in Ukraine’s One Health capacity and outlines opportunities to strengthen biosurveillance, laboratory systems, biosecurity, and interagency co-operation. The findings support Ukraine’s alignment with the European Union and global frameworks and contribute to a broader understanding of how the One Health model can be operationalized under conditions of armed conflict and resource limitation.

Despite the growing global recognition of the One Health approach as an essential framework for addressing zoonotic diseases, AMR, food security, and environmental degradation, its practical implementation remains uneven, particularly in conflict-affected settings. Most existing One Health literature focuses on stable or high-income contexts, while evidence from countries experiencing prolonged armed conflict is limited, fragmented, or descriptive. In Ukraine, although One Health principles have been formally adopted within national strategies and supported through international collaborations, there is a lack of comprehensive, integrative evaluations that critically assess how these principles function across human, animal, plant, and environmental health sectors under conditions of war.

Current studies in Ukraine predominantly address individual domains, such as zoonotic disease surveillance, AMR trends, veterinary public health, or environmental impacts, without systematically examining intersectoral linkages or governance mechanisms that define the One Health paradigm. Moreover, the environmental and plant health components of One Health remain underrepresented in both policy analyses and scientific assessments, despite their central role in food security, ecosystem resilience, and long-term population health. The extent to which war-related disruptions, including infrastructure destruction, workforce displacement, surveillance breakdowns, and funding instability, undermine multisectoral coordination has not been sufficiently analyzed within a unified One Health framework.

Additionally, there is limited synthesis of how national legislation, institutional arrangements, scientific programs, and international standards interact to shape One Health capacity in Ukraine. Existing reports often provide valuable but isolated insights, lacking a holistic perspective that integrates policy, practice, and empirical evidence. Consequently, critical gaps persist in understanding the structural, operational, and systemic barriers to effective One Health implementation, as well as the opportunities for strengthening resilience, sustainability, and alignment with European Union and global health security frameworks.

This scientometric review aims to characterize the One Health approach, clarify the principles of intersectoral co-operation underlying its implementation, and evaluate the current status and future prospects of One Health in Ukraine [1, 10, 11, 20, 21].

The aim of this scientometric is to provide a comprehensive, evidence-based evaluation of the One Health approach in Ukraine by integrating human, animal, plant, and environmental health perspectives within a single analytical framework. Specifically, this study seeks to characterize the conceptual foundations and evolution of the One Health approach, examine its incorporation into Ukraine’s national policies, research initiatives, and institutional structures, and assess its current implementation status in the context of ongoing armed conflict.

This scientometric further aims to identify key strengths, gaps, and challenges affecting One Health operationalization in Ukraine, with particular attention to multisectoral governance, disease surveillance systems, AMR, biosecurity, integration of environmental and plant health, and laboratory and workforce capacity. By synthesizing international standards, national legislation, scientific evidence, and practical case examples, the study seeks to highlight opportunities to improve intersectoral co-operation and strengthen national resilience.

Ultimately, this scientometric aims to contribute to national and international discourse by providing insights into how the One Health model can be adapted and sustained in conflict-affected and resource-constrained settings. The findings are intended to inform policymakers, researchers, and international partners, supporting the development of more effective, coordinated, and resilient One Health strategies in Ukraine and comparable contexts.

## MATERIALS AND METHODS

### Ethical approval

This study was based exclusively on the analysis of published scientific literature, publicly available policy documents, and secondary data obtained from international organizations and official institutional sources. No human participants, animals, or biological samples were directly involved, and no personal, identifiable, or confidential data were accessed or analyzed. Therefore, ethical approval from an institutional ethics committee or animal care and use committee was not required for this scientometric review, in accordance with national and international guidelines for research involving secondary data and document-based analyses.

### Study period and location

The scientometric review was conducted between June and August 2025. Bibliometric data were retrieved from the Scopus database and complemented by a review of publicly available policy documents, national legal frameworks, and reports from international organizations. Although the literature analyzed originated from multiple countries, the analytical focus of this study was Ukraine, with particular emphasis on research, policy, and implementation of the One Health approach in the context of the ongoing armed conflict.

### Study design

This study adopted a scientometric review design complemented by qualitative contextual analysis. The approach combined quantitative mapping of published scientific literature with purposive examination of policy documents, case studies, and secondary data sources. This mixed-method design was selected to characterize research trends, thematic distributions, and implementation challenges related to the One Health approach in Ukraine, particularly under conditions of armed conflict.

### Data source and literature search strategy

Bibliometric data were retrieved from the Scopus database, selected for its broad coverage of peer-reviewed international literature across health, environmental, agricultural, and interdisciplinary sciences. The literature search was conducted between June and August 2025. Search queries combined keywords related to the war in Ukraine, One Health, antimicrobial resistance, zoonotic diseases, food security, and environmental or ecological safety. The search strategy aimed to capture publications addressing at least one core One Health domain in the context of conflict, geopolitical disruption, or health system stress.

### Document selection and dataset composition

Publications retrieved from the database were screened at the title and abstract levels to assess thematic relevance. Records were retained if they addressed human, animal, plant, or environmental health and were relevant to war-associated impacts, biosecurity challenges, or disruptions of health, veterinary, or food systems. Publications focusing exclusively on unrelated political, economic, or non-health topics were excluded.

The final dataset used for the preliminary scientometric analysis comprised 548 publications, including 69 on zoonotic diseases, 397 on food security, 47 on environmental or ecological safety, and 35 on AMR. These records formed the basis for a descriptive assessment of publication volume and thematic distribution.

**Figure 1:**
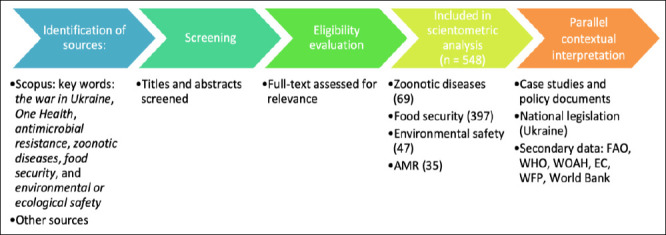
Flow diagram illustrating the identification, screening, eligibility assessment, and inclusion of publications for the scientometric analysis, together with complementary case studies and policy documents used for contextual interpretation.

To enhance transparency in dataset construction, the processes of record identification, screening, and inclusion are summarized in a structured flow diagram (Figure 1). This diagram illustrates the stages of literature retrieval, relevance screening, eligibility assessment, and final inclusion without implying a systematic review methodology.

### Case study and legal document selection

To complement the bibliometric findings and address limitations of gray literature, a purposive selection of case studies and policy documents was conducted. Case studies were selected to deepen the analysis of the practical impact of the war on One Health implementation in Ukraine, ensuring thematic relevance, scope diversity, and international significance. National legal and regulatory documents related to biosafety, biosecurity, public health, veterinary services, and environmental protection were retrieved from the official National Parliament portal of Ukraine (https://zakon.rada.gov.ua/laws/main/index) [17–19].

### Secondary data sources

Secondary data were reviewed to contextualize the impact of the war on food availability and food security. Reports and datasets were obtained from international organizations, including the Food and Agriculture Organization of the United Nations (FAO), World Health Organization (WHO), World Organization for Animal Health (WOAH), European Commission, World Food Program, and World Bank, as well as relevant Ukrainian statistical sources [10, 11, 13, 22–25]. These data were used to support the interpretation of trends related to grain production, supply chain disruption, and food system resilience within the One Health framework.

### Data synthesis and analysis

Scientometric data were analyzed descriptively to assess publication volume and thematic focus across One Health domains. Findings from the bibliometric analysis were integrated with evidence from case studies, national legislation, and secondary data sources to provide a contextualized overview of One Health implementation in Ukraine. The synthesis focused on identifying structural gaps, operational challenges, and opportunities to strengthen multisectoral collaboration in conflict-affected, resource-constrained settings.

## RESULTS AND DISCUSSION

### Conceptual foundations of the One Health approach

The One Health concept is a multidisciplinary, integrative framework that recognizes the intrinsic interconnection among human, animal, and environmental health. It emphasizes that optimal health outcomes can be achieved only through coordinated collaboration across multiple sectors, including human medicine and public health, veterinary medicine, ecology, and the biological sciences [25, 26]. The philosophy of One Health has evolved over centuries, reflecting both scientific advances and growing societal awareness of shared health determinants across species and ecosystems.

### Historical evolution of One Health

#### Early environmental health perspectives

The origins of the One Health concept can be traced back to ancient Greece. Hippocrates (~400 BC) emphasized the influence of environmental factors on human health in his treatise On Airs, Waters, and Places, asserting that physicians must consider climate, seasonal variation, water quality, and soil characteristics to practice effective medicine [27]. This early recognition of environmental determinants established the conceptual foundation for a holistic understanding of health that transcends individual species.

#### Zoonoses and the integration of human and animal medicine

In the 19th century, German scientist Rudolf Virchow (1821–1902) further advanced integrative health thinking through his work on zoonotic diseases, particularly Trichinella spiralis in pork. Virchow introduced the term “zoonosis” and famously stated that “there are no dividing lines between animal and human medicine, nor should there be” [26]. His contributions highlighted the need to address both human and animal populations in effective public health interventions.

#### Development of the “One Medicine” concept

The integrative approach was further strengthened by Canadian physician Sir William Osler, who taught both medical students at McGill College and veterinary students at the Montreal Veterinary College during the 1870s [28]. Osler promoted comparative pathology and advocated a unified medical approach, reinforcing the interdependence of human and veterinary medicine.

In the mid-20th century, veterinarians played a central role in applying the “One Medicine” framework within public health practice [26]. This vision was later expanded by Calvin Schwabe, who formally articulated the concept of “One Medicine” and advocated comprehensive integration of human and veterinary health strategies to address public health challenges [26, 28].

### Transition from One Medicine to One Health

The evolution from “One Medicine” to “One Health” reflected a broader and more preventive understanding of health, emphasizing the interconnectedness of humans, animals, and the environment. Contemporary One Health initiatives aim to improve ecosystem health and address emerging global challenges, including zoonotic disease outbreaks, AMR, and the health impacts of climate change [16, 29–32].

### One Health in the Ukrainian context

#### Policy framework and institutional development

In Ukraine, the One Health approach is currently in a developmental and institutionalization phase. National and public institutions have begun implementing integrative strategies that link human, animal, and environmental health in alignment with international standards for zoonotic disease control, veterinary public health, and ecosystem management [16, 20, 26].

In the Ukrainian context, One Health is understood as an operational framework that integrates human health, veterinary medicine, agriculture, and environmental protection to address zoonotic diseases, AMR, food safety, ecological risks, and biosecurity. This integration is reflected in national strategic documents, including the Strategy for Biosafety and Biosecurity, based on the One Health principle, for 2022–2025 [19], and the Healthcare System Development Strategy 2030 [18].

#### Capacity-building and policy alignment

Capacity-building initiatives support One Health implementation in Ukraine, notably through training courses jointly organized by the FAO, WHO, and partner organizations [1, 13, 16, 33] and the publication of the first Ukrainian One Health Manual in 2019 [34]. At the policy-level, the One Health approach serves as a guiding framework for strengthening biosecurity, informing legislative development (e.g., the draft Law on Biological Safety and Biological Security), and aligning Ukrainian health, veterinary, and environmental systems with European Union and global standards [22, 30, 35, 36]. Table 1 summarizes key policy and professional documents that support One Health implementation in Ukraine.

**Table 1:** Definitions of One Health in the Ukrainian legal framework and professional documents.

Document/Source	What it demonstrates/supports	How it fits into the definition
Cabinet of Ministers of Ukraine. (2022). Biosafety and biosecurity strategy for 2022–2025. Ministry of Environmental Protection and Natural Resources of Ukraine. Available from: https://mepr.gov.ua/en/uryad-vyznachyv-yak-vykonuvatymut-strategiyu-biobezpeky-ta-biologichnogo-zahystu-u-2022-2025-rokah	It shows that Ukraine has a national strategy for biosafety and biosecurity. Policy-level recognition of risks from pathogens and biological threats	supports your statement that One Health involves policy tools for biosecurity and risk monitoring.
Cabinet of Ministers of Ukraine. (2019). Order No. 1416-r: Strategy for biosafety and biosecurity based on the “One health” principle until 2025. Retrieved from https://uareforms.org/en/monitoring/oxorona-zdorovya	Confirms that Ukraine officially uses the term “One Health-adjacent” in its national strategy and has committed to a unified system of biosafety and biosecurity.	Useful citation to show “One Health” is not just academic but embedded in strategy and legislation drafting.
Food and Agriculture Organization of the United Nations. (2025, September 16). Strengthening Ukraine’s health systems through online One Health training. Available from: https://www.fao.org/countryprofiles/news-archive/detail-news/ru/c/1740200	Capacity-building in the One Health framework: cross-sectoral education/training in Ukraine	supports your statement that, in practice, One Health involves the training of professionals across sectors
One Health Manual (2019). NGO “One Health Institute.” https://www.onehealthinstitute.org.ua/publications	Shows that there is a “manual” of One Health in Ukraine, suggesting that local guidelines or definitions are tailored/translated /adapted	This information supports your claim that One Health is being adapted in the Ukrainian context (science, policy, education)
Translation of CDC’s Biosafety in Microbiological and Biomedical Laboratories, 6th edition. NGO “One Health Institute.” https://www.onehealthinstitute.org.ua/2025/03/15/the-private-scientific-institution-one-health-scientific-and-research-institute-and-the-ngo-one-health-institute-present-an-original-ukrainian-translation-of-the-cdc-manual-biosafety-in-micr	Institutional work on laboratory biosafety, standardization, regulation, and legislative initiatives	Bolster the operational part of your definition about “biosafety, biosecurity, regulation of labs, pathogens, etc.”
Cabinet of Ministers of Ukraine. (2021). Healthcare system development strategy 2030. Available at: https://healthstrategy2030.com.ua/en/strategy	Provides a broader strategic framework for health system reform, resilience, universal health coverage, and likely alignment with One Health principles (e.g., surveillance, capacity, and integrated health)	It is useful to say that One Health is aligned with Ukraine’s long-term national health strategy

CDC = Centers for Disease Control and Prevention, EU = European Union, FAO = Food and Agriculture Organization of the United Nations, NGO = Non-governmental organization, UNEP = United Nations Environment Programme, WHO = World Health Organization, WOAH = World Organisation for Animal Health.

### Current challenges and structural components

Ukraine faces multiple interrelated challenges, including zoonotic disease outbreaks, environmental degradation, intensive agricultural production, and emerging AMR, all of which require a comprehensive, multisectoral health framework.

**Figure 2:**
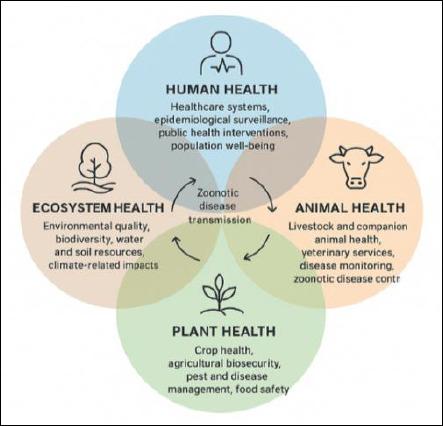
One Health Core Components in Ukraine (human, animal, and plant health).

According to the updated definition proposed by the One Health High-Level Expert Panel, Ukraine’s One Health model comprises four core components: human health, animal health, plant health, and environmental health. These components are closely linked and interdependent, forming an integrated system that sustainably balances and optimizes the health of people, animals, and ecosystems [16]. Figure 2 illustrates these core components in the Ukrainian context.

Key agencies, stakeholders, and their functional interactions within the One Health framework are presented in Table 2. These institutions collectively span all four One Health sectors, underscoring the need for coordinated multisectoral governance.

**Table 2:** Stakeholders and Functions in One Health System in Ukraine.

Agency/Organization	Network/Affiliations	Functions	Intersectoral Interactions
Ministry of Health	PHC; National Reference Laboratories	National public health policy; epidemiological surveillance; outbreak investigation; AMR stewardship (ECDC, 2023; WHO, 2023)	Coordinates with the SSUFSCP for zoonoses; exchanges surveillance data with environmental agencies; cooperates with the SESU during biological/chemical incidents
State Service on Food Safety and Consumer Protection (SSUFSCP)	National veterinary laboratories; SPS system; border inspection	Oversees animal health surveillance, food safety control, zoonotic disease detection, and plant health measures (FAO, 2023; WOAH, 2022)	Collaborates with the MoH on zoonotic outbreaks; coordinates with the MAPF on agriculture; and interacts with customs and border services
Ministry of Environmental Protection and Natural Resources, Japan	Hydrometeorological Service, State Environmental Inspectorate	Environmental monitoring, pollution assessment, biodiversity protection, and evaluation of war-related environmental risks (UNEP, 2022)	Shares environmental alerts with MoH and SSUFSCP; coordinates emergency assessments with SESU
Ministry of Agrarian Policy and Food	Agricultural research institutes; National Academy of Agrarian Sciences (NAAS)	Agricultural and plant health policy; pesticide regulation; oversight of food production system (FAO, 2023)	Works with the SSUFSCP for plant and animal health; coordinates with the MoH on foodborne risks
The State Emergency Service of Ukraine	Civil protection units and CBRN response teams	Responds to chemical, biological, and radiological events, environmental remediation, and disaster risk-reduction (World Bank, 2023)	Collaborates with all sectors during emergencies, especially MoH and environmental agencies
Ministry of Education and Science (MESU)	Universities and research institutes	Workforce development, epidemiologists, veterinarians, and ecologists training, and One Health academic programs (Rüegg et al., 2018)	Cross-sector education and research programs with medical, veterinary, and environmental institutions
International Organizations (WHO, FAO, WOAH, UNEP, ECDC, USAID, and EU)	Global OHJPA (One Health Joint Plan of Action) and regional networks	Technical assistance, funding, capacity-building, AMR surveillance systems, and laboratory strengthening (FAO *et al*., 2022; WHO, 2023)	Multisectoral coordination and harmonization of Ukrainian surveillance with international standards
Civil society and non-governmental organizations	Animal welfare groups; environmental non-governmental organizations; public health advocacy organizations	Implementation of community-level zoonotic prevention, stray animal vaccination, environmental monitoring, and health education (Sweileh, 2021)	Collaborating with local authorities, veterinary stations, and environmental inspectors

MoH = Ministry of Health, PHC = Public Health Center, AMR = Antimicrobial resistance, SSUFSCP = State Service of Ukraine for Food Safety and Consumer Protection, SPS = Sanitary and phytosanitary, MAPF = Ministry of Agrarian Policy and Food, NAAS = National Academy of Agrarian Sciences, SESU = State Emergency Service of Ukraine, CBRN = Chemical, biological, radiological, and nuclear, WHO = World Health Organization, FAO = Food and Agriculture Organization of the United Nations, WOAH = World Organisation for Animal Health, ECDC = European Centre for Disease Prevention and Control, UNEP = United Nations Environment Programme, NGO = Non-governmental organization.

#### Multisectoral Co-operation and Interactions within the One Health Framework

The One Health model emphasizes the interdependence of four core domains: human, animal, plant, and environmental health. In Ukraine, several critical multisectoral interactions demonstrate the practical relevance of this integrated framework.

#### Zoonotic diseases

Human encroachment into natural habitats and the intensification of farming systems facilitate the transmission of zoonotic pathogens, including avian influenza viruses, rabies virus, and Leptospira spp. [16]. These interactions increase contact among humans, domestic animals, wildlife, and contaminated environments, thereby increasing the risk of disease spillover.

#### AMR

The widespread use of antibiotics in both human medicine and livestock production accelerates the emergence and spread of antimicrobial-resistant pathogens, posing significant challenges for human and veterinary healthcare systems alike [37].

#### Climate change impacts

Alterations in temperature and precipitation patterns associated with climate change affect crop yields, influence vector population dynamics, and increase the incidence of waterborne diseases [15].

#### Biodiversity loss

Habitat fragmentation and environmental pollution reduce ecosystem resilience and increase the frequency of disease outbreaks [2].

Collectively, Ukraine’s One Health approach strengthens disease surveillance, promotes environmental sustainability, and improves public health outcomes. Multisectoral collaboration remains essential to prevent emerging infectious diseases, combat AMR, and mitigate the impacts of climate change and biodiversity loss.

### International support and One Health implementation initiatives

The One Health approach has been implemented in Ukraine with substantial support from international partners, including FAO, USAID, and WOAH, through initiatives such as African swine fever (ASF) STOP (COST Action) [38], PREDICT [39], READY [40], and the One Health Workforce programs [41, 42].

A dedicated project, supported by a grant from the US Civilian Research and Development Foundation (CRDF Global) and funded by the US State Department, was implemented to develop a national plan for One Health implementation. Within this framework, three interagency One Health meetings and workshops were held in Madrid in April 2017 and in Odessa in September 2017 and 2018 [34].

### National policy and legislative framework for One Health

At the state level, the One Health approach is implemented in accordance with the Order of the Cabinet of Ministers of Ukraine dated November 27, 2019 (No. 1416-r), which approved the Strategy for Ensuring Biological Safety and Biological Security based on the One Health principle for the period up to 2025, along with its associated action plan [17]. The strategy was developed in the context of the Association Agreement between Ukraine and the European Union, the European Atomic Energy Community, and their Member States. The Agreement was ratified by Law No. 1678-VII on September 16, 2014.

According to an FAO report on Ukraine, “One Health has become a cornerstone of zoonosis prevention in Ukraine, particularly in addressing ASF, rabies, leptospirosis, and AMR” [13].

### Risk prioritization and One Health capacity-building

In 2021, with support from the Centers for Disease Control and Prevention and the WHO, a workshop was held to prioritize animal and human pathogens using the One Health approach. The objectives of the workshop were to prioritize zoonotic diseases of greatest concern in Ukraine in accordance with the International Health Regulations (IHR, 2005) and to develop subsequent action plans in collaboration with international partners [21, 25, 43].

Following this prioritization exercise, a WHO seminar on implementing the Joint Risk Assessment Tool was held [1, 15, 33]. During the seminar, scientists and practitioners from public health and veterinary medicine assessed the risks of tularemia, rabies, and leptospirosis spreading in Ukraine under martial law.

Between 2019 and 2022, within the framework of the Organization for Security and Cooperation in Europe biosafety project, a draft Law of Ukraine titled “On the System of Biological Safety and Biological Security in Ukraine” was prepared, along with several subordinate legislative acts [1, 17]. A joint working group on biological legislation was established and conducted the reclassification of biological agents in line with international requirements. National biosafety standards were also developed during this period.

### Surveillance systems and institutional infrastructure

**Figure 3:**
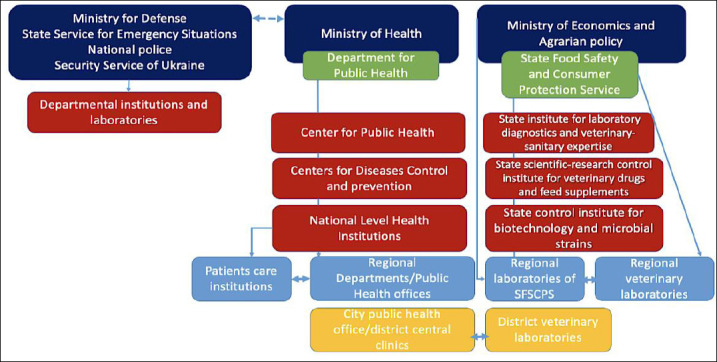
Epidemiological surveillance system in Ukraine: Intersectoral co-operation between the MoH and SSUFSCP.

Zoonotic and emerging disease surveillance and diagnostic activities in Ukraine are conducted by multiple scientific and scientific-practical institutions. These institutions operate under the Ministry of Health of Ukraine, the State Service of Ukraine for Food Safety and Consumer Protection, the National Academy of Agrarian Sciences, and the National Academy of Medical Sciences of Ukraine (Figure 3) [34].

The Public Health Center of the Ministry of Health maintains a network of 25 regional Centers for Disease Control, which are responsible for infectious disease surveillance, AMR monitoring, and chemical, biological, radiological, and nuclear (CBRN) emergency management. On the animal health side, the Scientific and Research Institute for Laboratory Diagnostics and Veterinary and Sanitary Expertise of the State Service of Ukraine for Food Safety and Consumer Protection, together with a network of 25 regional laboratories, conducts surveillance of animal infectious diseases, zoonoses (in co-operation with the Ministry of Health network), AMR, pesticide and toxicant residues, and food safety and security.

### Research projects and international biosecurity programs

The institutions described above have implemented several major scientific projects, including UP-4 (risk assessment of selected especially dangerous pathogens potentially carried by migratory birds over Ukraine, 2016–2017), UP-9 (analysis of ASF virus transmission in domestic pigs and wild boar using genome sequencing and phylogenetic analysis, 2017–2018), and UP-10 (regional field-to-table risk assessment of ASF spread across Ukraine via wildlife and consumer trade routes, 2018–2020) [44].

Additional support has been provided through the German Biosecurity Program, including the German–Ukrainian Biosecurity Initiative for Zoonosis Risk Management Near the External EU Border (2021–2025) and OSCE-funded projects to establish sustainable veterinary surveillance systems for especially dangerous pathogens (2022–2024).

### Quantitative evidence on the burden of zoonotic diseases in Ukraine

Available data underscore the significant burden of zoonotic diseases in Ukraine and highlight the need for an integrated One Health approach.

Leptospirosis

Leptospirosis, a bacterial zoonotic disease transmitted through contact with contaminated water or soil, has been reported in Ukraine. In 2023, the human leptospirosis notification rate reached 1.06 per 100,000 population, exceeding rates reported in many European Union countries [45]. This finding indicates a substantial public health concern and the need for enhanced surveillance and control measures. However, disease incidence and surveillance data remain complex because infections in natural foci cannot be fully controlled [46–49].

#### Rabies

Rabies remains a major zoonotic threat in Ukraine. Human deaths were reported in both 2023 and 2024, highlighting persistent risks despite ongoing control efforts [45]. War-related ecological changes, including increased rodent populations, mouse migration into human habitats, and unharvested crops during 2022–2023, contributed to an increase in red fox populations. The absence of adequate oral vaccination campaigns has led to increased rabies cases in both animals and humans [50–54].

#### Foodborne zoonoses

Intensive animal farming operations in Ukraine have been linked to bacterial zoonotic disease outbreaks, particularly salmonellosis and campylobacteriosis [29, 45]. These outbreaks are associated with industrial poultry and livestock farms, where production conditions may facilitate pathogen transmission to humans. Improving farm biosecurity remains essential. Several risk-based initiatives, including projects on live vaccine safety and milk quality, have contributed to roadmaps for improving animal product safety, and their outcomes have been implemented under the supervision of the State Service of Ukraine for Food Safety and Consumer Protection.

#### Brucellosis

Brucellosis remains a public health concern in Ukraine. Although comprehensive incidence and prevalence data are limited, the persistence of the disease in animal populations continues to pose ongoing risks, particularly in rural areas [45, 53].

#### Tuberculosis (TB) and other major infectious diseases

Ukraine continues to bear a substantial burden of TB. In 2023, the estimated TB incidence rate reached 112 cases per 100,000 population, a 13% increase over the previous year [55]. This trend underscores an ongoing public health challenge that requires strengthened surveillance and intervention strategies.

Regional variation in TB incidence is pronounced. In Central Ukraine, incidence increased from 36.55 per 100,000 in 2020–2021 to 62.75 per 100,000 in 2022–2023 [53, 55], whereas Eastern Ukraine saw a decline over the same period. These differences underscore the need for region-specific public health responses.

Multidrug-resistant (MDR) and rifampicin-resistant TB remain critical challenges. In 2022, approximately 29% of new TB cases and 43% of previously treated cases were classified as MDR/RR-TB [55]. This high prevalence underscores the need for advanced diagnostics and effective treatment regimens. Although Ukraine-specific mortality data are limited, TB remains one of the leading infectious disease causes of death globally, with an estimated 1.25 million deaths worldwide in 2023 [21, 55].

The ongoing conflict has exacerbated TB transmission by disrupting healthcare services and limiting access to treatment, contributing to increased incidence and complicating the management of drug-resistant cases [31–33, 55]. These factors underscore the importance of resilient health systems.

#### Vector-borne and respiratory diseases

Vector-borne diseases in Ukraine include Lyme borreliosis, tick-borne encephalitis, and West Nile virus infection [48, 53, 56–59]. Surveillance of these diseases is increasingly challenged by war-related disruptions and landmine contamination, which restrict access to natural foci and complicate entomological sampling.

Seasonal influenza remains a significant respiratory disease, causing substantial morbidity and occasional mortality, particularly among high-risk populations [33]. Approximately 1.2 million influenza-like illness cases were reported nationwide during the 2022–2023 season, with several hundred severe cases requiring hospitalization [53, 60]. Vaccination coverage remains low, with less than 5% of the population vaccinated in recent seasons [33].

### Economic impact of zoonotic diseases

Zoonotic diseases impose a substantial economic burden on livestock production in Ukraine. Diseases such as brucellosis, TB, and mastitis cause direct losses through reduced productivity and higher veterinary costs, as well as indirect losses from trade restrictions and public health interventions [31, 32].

Although available data demonstrate significant public health and economic impacts, they remain insufficient for comprehensive risk assessment and evidence-based policy formulation. Therefore, enhanced surveillance, improved data integration, and strengthened intersectoral collaboration are urgently required.

### AMR: surveillance, trends, and challenges

Ukraine has made notable progress in strengthening its AMR surveillance infrastructure. With WHO support, the Central Asian and European Surveillance of Antimicrobial Resistance (CAESAR) network expanded to include 67 laboratories across 22 regions by 2023, all of which adhered to European Committee on Antimicrobial Susceptibility Testing methodologies [21, 33, 61, 62].

Nevertheless, high resistance rates to third-generation cephalosporins and carbapenems among Gram-negative bacteria, including Klebsiella pneumoniae and Escherichia coli, remain a major concern [59]. This conflict has further intensified AMR challenges, with increased detection of MDR and Extensively drug-resistant organisms, including NDM-1-producing K. pneumoniae, in both civilian and military healthcare settings [63, 64].

Destruction of healthcare infrastructure, with more than 1,700 facilities damaged or destroyed by July 2024, has further constrained AMR surveillance and control efforts [1, 21]. International collaborations, particularly with the Centers for Disease Control and Prevention, continue to support initiatives in laboratory strengthening, infection control, and antimicrobial stewardship [63, 65].

### Plant health and environmental risks

#### Mycotoxins and crop pests

Mycotoxins such as deoxynivalenol and aflatoxins contaminate cereals and pose risks to human and animal health in Ukraine [66–70]. Crop pests also significantly affect agricultural productivity, and chemical pesticides are commonly used to prevent yield losses of up to 33%–48% [71]. However, overreliance on chemical pesticides has contributed to pest resistance and environmental contamination, prompting interest in biopesticides as alternative solutions [68].

#### Pesticide and antimicrobial use in agriculture

The ongoing conflict disrupted supply chains for plant protection products in 2022, reducing availability, yet chemical pesticide use remains prevalent [71]. Concerns persist about pesticide toxicity, groundwater contamination, and long-term soil persistence [23, 68]. The use of antimicrobials in crop production is an emerging issue, with global evidence indicating that misuse contributes to AMR affecting plant, animal, and human health [12, 13].

#### Environmental pollution and health outcomes

Environmental pollutants, including sulfur dioxide, particulate matter, and non-methane volatile organic compounds, have been linked to hormonally mediated and urban cancers in Ukraine. Conflict-related infrastructure damage, population displacement, and underreporting further shape geographic disparities. Spatial analyses and heatmaps help identify recurrent pollution-cancer associations, underscoring the need for integrated environmental monitoring, strengthened diagnostic capacity, and regionally tailored public health strategies [72].

#### Funding sources supporting One Health activities in Ukraine

The implementation and sustainability of One Health activities in Ukraine depend on a mix of domestic and international funding sources. The Ukrainian government has allocated resources to strengthen public health infrastructure, particularly in priority areas such as AMR, zoonotic disease surveillance, and integrated health systems [6, 23].

In addition to governmental support, non-governmental organizations (NGO) play a pivotal role in advancing One Health research, monitoring, and prevention strategies across the human, animal, and plant health sectors. A series of measures to implement the One Health approach has been introduced through public initiatives led by the Private Scientific Institution “One Health Scientific and Research Institute” and the NGO One Health Institute (https://www.onehealthinstitute.org.ua/uk/836-2/).

International support has been instrumental in complementing domestic funding and expanding the scope of One Health initiatives. The WHO has provided substantial technical and financial assistance during the ongoing crisis, including US$240 million in emergency funding in 2023 [62]. The Centers for Disease Control and Prevention collaborates with the Ukrainian Ministry of Health to enhance surveillance systems, strengthen antimicrobial stewardship, and support zoonotic disease control programs [11, 20]. The European Commission, through Horizon Europe initiatives, funds research projects addressing AMR and promoting integrated One Health approaches. The Food and Agriculture Organization supports interventions to mitigate zoonotic and food safety risks within the broader One Health framework [13]. Additionally, the Global Fund and the World Bank provide targeted funding to maintain health services and provide sectoral support. In 2023, the Global Fund approved US$27.7 million for HIV and TB prevention, testing, and treatment services, while the World Bank committed US$2.05 billion to development and health-sector support in Ukraine [23].

### Practical case studies and lessons learned from One Health implementation

#### ASF

Despite growing recognition of One Health principles in Ukraine, detailed case studies of interventions remain limited. ASF is a key example. Since its introduction in 2012, ASF has caused substantial economic losses in the Ukrainian swine industry. Coordinated interventions by the State Service of Ukraine for Food Safety and Consumer Protection and regional veterinary laboratories, including surveillance, culling of affected herds, and enforcement of biosecurity measures, demonstrated the importance of multisectoral coordination among veterinary services, local authorities, and farmers [13]. However, reporting delays and limited public awareness in some regions facilitated further spread, highlighting gaps in communication and community engagement [44].

#### Rabies vaccination campaigns

In Ukraine, coordinated rabies vaccination campaigns targeting domestic animals and wildlife reservoirs have improved disease control. Integrating public health authorities, veterinary services, and community stakeholders led to a measurable reduction in human rabies cases over the past decade [62]. However, the war created substantial challenges, and full-scale wildlife vaccination was restored only in 2024. Two missed vaccination seasons led to outbreaks among wildlife and domestic animals and increased fatal human cases. International experts have identified that these risks continue to grow [50].

#### Coronavirus disease 2019 (COVID-19) multisectoral response

The COVID-19 pandemic underscored the importance of integrated health approaches. Ukraine established coordination mechanisms among human health authorities, veterinary laboratories capable of SARS-CoV-2 diagnostics, and environmental monitoring units. This collaboration enabled enhanced surveillance of zoonotic spillover risks and supported timely public health responses [20]. Nevertheless, limited resources and uneven interagency communication hindered optimal outcomes, underscoring the importance of pre-established One Health networks.

### Critical analysis of One Health domains in Ukraine

#### Policy and governance challenges

Despite the formal adoption of the One Health framework within national strategies, Ukraine’s governance structure remains fragmented. Sectoral ministries continue to operate under separate mandates without institutionalized coordination mechanisms, reflecting a broader global challenge in translating One Health policy into operational practice [16, 73]. Overlapping responsibilities among health, veterinary, agricultural, and environmental authorities impede timely decision-making and weaken accountability. Donor-driven initiatives address critical gaps but create reliance on external priorities, reducing national ownership. As a result, the policy environment demonstrates conceptual commitment but lacks regulatory integration, budget allocation, and long-term governance infrastructure for sustainable One Health implementation [57].

#### Surveillance system limitations

Ukraine’s human, animal, and plant health surveillance systems remain only partially interoperable, reflecting structural weaknesses common in many low- and middle-income countries [52]. Parallel platforms hinder real-time data exchange and delay detection of cross-sectoral threats. War-related laboratory destruction, territorial loss, and population displacement have further degraded surveillance capacity [42, 63, 74]. Although rabies and ASF surveillance systems have shown resilience, overall digital harmonization and laboratory support remain inadequate.

#### AMR

AMR is one of the most critical cross-sector threats to Ukraine’s health security. Conflict conditions have accelerated AMR emergence through increased empirical antibiotic use, weakened infection prevention and control, and diagnostic shortages [5]. Surveillance shows rising resistance rates in hospitals, while veterinary AMR monitoring remains inconsistent and poorly regulated [75]. Regulatory gaps in livestock antimicrobial use and limited stewardship training undermine national AMR governance. Although Ukraine participates in international AMR networks such as CAESAR, war-related strain and resource shortages hinder implementation of the national AMR action plan [63].

#### Zoonotic disease risks

Zoonotic diseases, including rabies, brucellosis, leptospirosis, and vector-borne infections—remain endemic in Ukraine. These risks are intensified by disrupted veterinary services, reduced vaccination coverage, and conflict-driven ecological changes [45]. Wildlife migration patterns and expansion of stray animal populations further increase exposure risks. Despite extensive experience in zoonotic disease control, Ukraine lacks integrated human–animal–environment data systems capable of predictive risk modeling [39]. Surveillance and response remain largely reactive.

#### Environmental and plant health gaps

Environmental and plant health remain the least developed components of Ukraine’s One Health system. Despite their relevance to food security, pollution-related illness, and ecosystem resilience, these sectors receive limited funding and political attention. War-related soil and water contamination, along with biodiversity loss, increase the risk of disease emergence. Plant health challenges, including fungal contamination, mycotoxins, and invasive pests, threaten agricultural productivity and food security [13, 16]. However, environmental and plant health data are rarely integrated into public health or veterinary decision-making.

#### Capacity and infrastructure constraints

Operationalizing One Health requires a trained workforce, adequate laboratory infrastructure, and stable funding. Ukraine faces shortages of epidemiologists, veterinarians, laboratory diagnosticians, and environmental health specialists, a trend observed globally where multidisciplinary training is limited [57]. Conflict-related workforce displacement and the destruction of laboratories, particularly BSL-3 facilities, have undermined surveillance, outbreak response, and AMR management. Dependence on donor-funded equipment and reagents further destabilizes laboratory operations during supply chain disruptions.

### Conflict-associated impacts on One Health implementation

Armed conflict acts as a systemic disruptor that amplifies vulnerabilities across all One Health sectors. In Ukraine, war has damaged healthcare, veterinary, and environmental monitoring infrastructure; disrupted supply chains; displaced populations; and increased exposure to environmental hazards [23]. Overburdened hospitals have accelerated AMR transmission [63], while damaged water and sanitation systems increase infectious disease risks. Environmental contamination from industrial destruction, unexploded ordnance, and toxic waste creates long-term health threats that remain insufficiently monitored [57, 66].

Conflict imposes profound structural, economic, and institutional pressures that directly hinder One Health implementation. Evidence from other conflict-affected regions shows that war reduces surveillance capacity, disrupts disease reporting, and weakens laboratory infrastructure essential for zoonotic disease detection and AMR monitoring [73]. In Ukraine, targeted attacks on civilian infrastructure, territorial occupation, and restricted service delivery have intensified these challenges [63].

Financial instability further compounds systemic weaknesses as national budgets are reallocated toward military and emergency needs. Funding for surveillance systems, diagnostics, vaccination programs, and environmental monitoring becomes limited, reducing the capacity to implement integrated One Health activities [57]. Long-term budget deficits have resulted in economic losses estimated at hundreds of billions of dollars. International donors, including WHO, FAO, WOAH, USAID, and the EU, have become primary funding sources for essential One Health–related functions, though such support remains vulnerable to geopolitical shifts and donor fatigue.

Despite these constraints, Ukraine has demonstrated resilience by integrating One Health principles into its biosafety strategy, national AMR action plan, and veterinary service modernization. However, implementation remains uneven because of infrastructure losses, workforce shortages, fragmented data systems, and insufficient funding for laboratory reconstruction. Conflict-driven changes in wildlife movement, livestock abandonment, environmental contamination, and reduced access to preventive veterinary care further increase zoonotic spillover risks, as documented in other war-affected regions [73].

Overall, Ukraine’s experience illustrates how armed conflict and financial instability undermine the operationalization of One Health by weakening governance structures, disrupting multisectoral communication, limiting surveillance capacity, and preventing long-term investment in health security. Strengthening One Health in conflict-affected settings, therefore, requires sustained international financing, capacity-building initiatives, and post-conflict reconstruction strategies that explicitly integrate human, animal, and environmental health. Without such support, fragile systems will struggle to prevent emerging zoonoses, contain AMR, and monitor environmental degradation, threats that extend beyond national borders.

#### One Health action plan for Ukraine: short-, medium-, and long-term perspectives

The One Health approach recognizes the fundamental interconnectedness of human, animal, and environmental health. In Ukraine, challenges such as emerging zoonotic diseases, AMR, environmental degradation, and the ongoing impacts of armed conflict underscore the need for a coordinated, multisectoral response. This section outlines a proposed One Health Action Plan for Ukraine, organized around short-, medium-, and long-term priorities. The framework aligns with international guidance, including the One Health Joint Plan of Action (2022–2026) and the IHR, 2005, while incorporating national priorities in health security, capacity-building, and sustainable development.

### Governance and coordination

Effective One Health implementation depends on robust governance structures that enable intersectoral collaboration and data sharing [57]. In the short-term, Ukraine should establish a National One Health Steering Committee comprising representatives from the Ministry of Health, the Ministry of Agrarian Policy, the Ministry of Environmental Protection, veterinary services, academic institutions, and civil society organizations. This committee would coordinate multisectoral activities, review existing legal frameworks, and facilitate regular intersectoral meetings [19].

In the medium-term, governance efforts should focus on institutionalizing One Health functions within permanent government structures and regional coordination hubs to ensure continuity. Long-term governance strategies should prioritize legislative integration that mandates data sharing and collaborative decision-making across sectors, and align national policies with European Union regulations and international health standards [62].

### Disease surveillance and risk assessment

Ukraine faces persistent and emerging risks from zoonotic diseases such as rabies, avian influenza, and other emerging pathogens. Short-term priorities include conducting One Health Zoonotic Disease Prioritization workshops, mapping high-risk areas, and piloting Joint Risk Assessments to guide targeted interventions by FAO, WHO, and other partners. Medium-term objectives include scaling up integrated surveillance systems that link wildlife, livestock, and human health data and establishing multidisciplinary rapid response teams. Long-term goals include continuous risk mapping that integrates ecological, climatic, and demographic variables and the deployment of early warning systems to predict and prevent outbreaks [57, 62].

### Capacity-building and workforce development

A shortage of trained professionals across the human, veterinary, and environmental health sectors is a major barrier to One Health implementation in Ukraine. Short-term actions should expand existing training opportunities, including FAO-supported online One Health courses, and strengthen laboratory capacity for coordinated diagnostic testing.

Medium-term strategies include embedding One Health principles into university curricula and continuing professional development programs, as well as establishing research initiatives focused on priority health threats. Long-term objectives emphasize creating national or regional centers of excellence for One Health research and innovation, fostering interdisciplinary collaboration, and retaining skilled professionals through structured career pathways and professional networks [4].

### AMR and environmental health

AMR poses a significant threat to both human and animal health in Ukraine. The National Action Plan on AMR (2018) provides a foundation for integrated stewardship across human, veterinary, and environmental sectors. Short-term priorities include strengthening AMR surveillance and laboratory diagnostic capacity.

Medium-term measures focus on implementing antimicrobial stewardship programs in clinical and agricultural settings, monitoring environmental contamination, and promoting rational antimicrobial use. Long-term interventions emphasize sustained surveillance, research into alternative therapies, and integration of environmental risk management into AMR mitigation strategies [57].

### Community engagement and risk communication

Community participation is critical to the success of One Health interventions. Short-term strategies include public awareness campaigns targeting priority zoonoses and AMR, particularly in rural and conflict-affected areas. Medium-term approaches include participatory surveillance initiatives and community-based risk-reduction programs. Long-term strategies aim to institutionalize health education in schools and local communities, fostering a sustained culture of prevention and risk awareness [4].

### Financial support and sustainability

Sustainable financing is essential for long-term One Health implementation. In the short-term, Ukraine should leverage international donor funding and align investments with the National Action Plan for Health Security. Medium-term strategies include integrating One Health activities into national and sectoral budgets. Long-term objectives include establishing a dedicated One Health Fund to support continuous operations, research, and infrastructure development.

### Monitoring and evaluation

Monitoring and evaluation should be embedded across all phases of the One Health Action Plan. Key indicators include the frequency of multisectoral meetings, surveillance coverage, the number of trained personnel, community awareness levels, and budget allocations. A mid-term review at 3 years and a final evaluation at 10 years are recommended to support adaptive management and continuous improvement.

### Overall vision

The proposed One Health Action Plan provides a comprehensive roadmap to strengthen health security in Ukraine through coordinated human, animal, and environmental health interventions. By adopting phased, intersectoral strategies and investing in governance, surveillance, workforce development, and community engagement, Ukraine can enhance its capacity to prevent, detect, and respond to emerging health threats. Alignment with international frameworks, such as the One Health Joint Plan of Action and the IHR, ensures consistency with global best practices and supports national resilience and recovery.

## CONCLUSION

This scientometric review offers a comprehensive overview of the evolution, scope, and implementation of the One Health approach in Ukraine, with particular attention to how armed conflict affects human, animal, plant, and environmental health systems. By integrating quantitative mapping of the scientific literature with qualitative analysis of policy frameworks, institutional arrangements, and case-based evidence, the review highlights both progress achieved and persistent structural gaps in operationalizing One Health in a conflict-affected setting.

The scientometric findings show substantial growth in One Health–related publications linked to Ukraine since 2014, with dominant research themes focused on zoonotic diseases, food security, AMR, and public health preparedness. In contrast, environmental and plant health dimensions remain comparatively underrepresented, indicating an imbalance in the One Health knowledge base. This thematic skew reflects broader global trends but is particularly concerning in Ukraine, where war-related environmental degradation, agricultural disruption, and pollution pose growing risks to health security and ecosystem resilience.

Despite formal policy adoption and alignment with international frameworks, including the One Health Joint Plan of Action and the IHR, the review identifies significant challenges in translating strategic commitments into sustained practice. Fragmented governance structures, limited interoperability of surveillance systems, workforce shortages, damage to laboratory infrastructure, and heavy reliance on donor-driven initiatives continue to constrain multisectoral coordination. Armed conflict acts as a systemic stressor, amplifying existing vulnerabilities by disrupting health services, accelerating AMR, increasing zoonotic spillover risks, and degrading environmental monitoring capacity.

At the same time, the Ukrainian experience demonstrates notable resilience. Continued participation in international surveillance networks, implementation of biosafety and biosecurity strategies, development of national AMR action plans, and engagement with global partners demonstrate the country’s commitment to maintaining One Health functionality under extraordinary conditions. These efforts offer valuable lessons for other conflict-affected and resource-constrained countries seeking to sustain integrated health approaches.

From a methodological perspective, this scientometric review underscores the value of combining bibliometric analysis with policy and case-based evidence to capture the multidimensional nature of One Health implementation. However, limitations in database coverage, underrepresentation of gray literature, and disruptions in data reporting during wartime should be acknowledged when interpreting trends.

In conclusion, strengthening One Health in Ukraine requires moving beyond conceptual endorsement to institutionalized governance, integrated surveillance across sectors, sustained investment in workforce and laboratory capacity, and systematic inclusion of environmental and plant health components. Long-term resilience will depend on coordinated national leadership, stable financing, and continued international support explicitly aligned with One Health objectives. The findings of this review contribute to the global understanding of how the One Health framework can be adapted, sustained, and operationalized in conflict settings, offering insights relevant far beyond the Ukrainian context.

## DATA AVAILABILITY

The supplementary data can be made available from the corresponding author upon request.

## AUTHORS’ CONTRIBUTIONS

AG: Conceptualization, methodology, formal analysis, supervision, and writing - original draft, editing, and revision. NS: Conceptualization, methodology, formal analysis, and writing - original draft, editing, and revision. OP, MR, and HA: Informal provision, investigation, methodology, and formal analysis. IG: Formal analysis and writing and proofreading. OO: Investigation, methodology, formal analysis, and proofreading. All authors have read and approved the final version of the manuscript.

## References

[1] World Health Organization (2024). A vision for health for all by 2030:Ukraine country co-operation strategy.. WHO Regional Office for Europe.

[2] Zinsstag J, Schelling E, Waltner-Toews D, Tanner M (2011). From “one medicine”to “One Health”and systemic approaches to health and well-being. Prev Vet Med.

[3] Cui Y, Zhang H, Li X (2025). Water resources, security, and conflict:A global outlook. Environ Sustain Rev.

[4] Destoumieux-Garzón D, Mavingui P, Boetsch G, Boissier J, Darriet F, Duboz P, Fritsch C, Giraudoux P, Le Roux F, Morand S, Paillard C, Pontier D, Sueur C, Voituron Y (2018). The One Health concept:10 years old and a long road ahead. Front Vet Sci.

[5] Murray CJL, Ikuta KS, Sharara F, Swetschinski L, Aguilar GR, Gray A, Han C, Bisignano C, Rao P, Wool E, Johnson SC, Browne AJ, Chipeta MG, Fell F, Hackett S, Haines-Woodhouse G, Kashef Hamadani BH, Kumaran EAP, McManigal B, Naghavi M (2022). Global burden of bacterial antimicrobial resistance in 2019:A systematic analysis.. Lancet.

[6] EU4Business (2021). Ukrainian invention measures food safety. https://eu4business.org.ua/en/success-stories/ukrainian-invention-measures-food-safety..

[7] Danielsen S, Schaffner U, Zinsstag J (2025). Expanding One Health to agriculture:Integrating plant, animal, and human health.. One Health Adv.

[8] Danielsen S, Schaffner U, Zinsstag J (2025). Worlds apart:Plant health and One Health and a path to convergence. CABI One Health.

[9] Scholthof KBG (2024). Plant pathology and One Health:Toward a broader framework. Annu Rev Phytopathol.

[10] Centers for Disease Control and Prevention (2024). CDC global health:Ukraine. https://www.cdc.gov/global-health/countries/ukraine.html..

[11] Centers for Disease Control and Prevention (2024). Ukraine One Health overview. https://www.cdc.gov/one-health/media/pdfs/2024/05/Ukraine-508.pdf..

[12] FAO (2023). Mycotoxins:A silent risk to plants, people and animals. https://www.fao.org/one-health/highlights/mycotoxins--a-silent-risk-to-plants--people-and-animals/en..

[13] FAO (2025). Strengthening Ukraine's health systems through online One Health training. https://www.fao.org/countryprofiles/news-archive/detail-news/ru/c/1740200..

[14] Vasylyev M, Lamberink H, Svyst I, Khlypnyach O, Sluzhynska O, Sluzhynska M, Shtoiko I, Hrushynska O, Demianenko D, Rokx C (2024). The infectious disease burden among war related internally displaced people in the Lviv region of Ukraine. Germs.

[15] Lerner H, Berg C (2017). A comparison of three holistic approaches to health:One Health, EcoHealth, and Planetary Health. Front Vet Sci.

[16] FAO UNEP WHO WOAH (2022). One Health Joint Plan of Action (2022–2026):Working together for the health of humans, animals, plants and the environment. https://www.fao.org/documents/card/.

[17] Cabinet of Ministers of Ukraine (2019). Order No. 1416-r:Strategy for biosafety and biosecurity based on the one health principle until 2025. https://uareforms.org/en/monitoring/oxorona-zdorovya..

[18] Cabinet of Ministers of Ukraine (2021). Healthcare system development strategy 2030. https://healthstrategy2030.com.ua/en/strategy..

[19] Cabinet of Ministers of Ukraine (2022). Strategy for biosafety and biosecurity for 2022–2025.. Ministry of Environmental Protection and Natural Resources of Ukraine.

[20] Centers for Disease Control and Prevention (2025). Status and prospects of improvement of health care in Ukraine:Legal aspects.. https://www.cdc.gov/one-health/media/pdfs/2024/05/Ukraine-508.pdf..

[21] World Health Organization (2024). Ukraine –WHO Data. https://data.who.int/countries/804..

[22] European Commission (2024). Horizon Europe project:Strengthening One Health research in Ukraine. https://cordis.europa.eu/project/id/101160053..

[23] World Bank Government of Ukraine European Union United Nations (2025). Ukraine –Fourth rapid damage and needs assessment (RDNA4):February 24, 2022 –December 31, 2024. https://documents1.worldbank.org/curated/en/099022025114040022/pdf/P180174-ca39eccd-ea67-4bd8-b537-ff73a675a0a8.pdf..

[24] World Health Organization (2018). National action plan on antimicrobial resistance –Ukraine. https://www.who.int/europe/news/item/21-02-2018-ukraine-develops-national-action-plan-on-antimicrobial-resistance..

[25] World Health Organization (2021.). One Health. https://www.who.int/news-room/fact-sheets/detail/one-health.

[26] Centers for Disease Control and Prevention (2025). One Health history. https://www.cdc.gov/one-health/about/one-health-history.html..

[27] Hippocrates On airs, waters, and places. The Internet Classics Archive.

[28] Dictionary of Canadian Biography (2025). William Osler. https://www.biographi.ca/en/bio/Osler_william_12E.html..

[29] EMR Antimicrobial Resistance Collaborators (2025). The burden of bacterial antimicrobial resistance in the WHO Eastern Mediterranean Region 1990–2021:A cross-country systematic analysis with forecasts to 2050.. Lancet Public Health.

[30] European Commission (2022). Animal Health Law:EU Regulation 2016/429.. https://eur-lex.europa.eu/legal-content/EN/TXT/?uri=CELEX%3A32016R0429..

[31] GBD 2023 Causes of Death Collaborators (2025). Global burden of 292 causes of death in 204 countries and territories and 660 subnational locations, 1990–2023:A systematic analysis for the Global Burden of Disease Study 2023.. Lancet.

[32] GBD 2023 Demographics Collaborators (2025). Global age-sex-specific all-cause mortality and life expectancy estimates for 204 countries and territories and 660 subnational locations, 1950–2023:A demographic analysis for the Global Burden of Disease Study 2023.. Lancet.

[33] World Health Organization (2023). Strengthening health system capacity in Ukraine. https://www.who.int/europe/news/item/22-08-2023-strengthening-ukraine-s-amr-surveillance-with-who-support..

[34] Gerilovych AP (2019). One Health Manual. Kharkiv, Ukraine:Institute of Experimental and Clinical Veterinary Medicine.

[35] Law of Ukraine No 2573-IX. About the public health system. Verkhovna Rada of Ukraine.

[36] Ministry of Health of Ukraine (2019). Public Health Law of Ukraine. https://zakon.rada.gov.ua/laws/show/2804-19.

[37] Rwego IB, Isabirye-Basuta G, Gillespie TR, Goldberg TL (2016). One Health approaches to disease surveillance:The case of zoonoses in Africa. Curr Opin Environ Sustain.

[38] COST Action CA15116 (2020). ASF-STOP:Understanding and combating African swine fever in Europe. https://www.cost.eu/actions/CA15116/..

[39] PREDICT Consortium PREDICT project:Building global capacity for detection of zoonotic viruses and pandemic prevention.

[40] READY Initiative (2019). READY:Strengthening global readiness to respond to public health emergencies. https://www.ready-initiative.org..

[41] Reuters (2025). Ukraine aid groups cut services after US funding shock. https://www.reuters.com/world/europe/ukraine-aid-groups-cut-services-scramble-cash-after-us-funding-shock-2025-01-30.

[42] The Global Fund (2023). Ukraine. https://www.theglobalfund.org/en/ukraine/..

[43] Stärk KD, Arroyo Kuribreña M, Dauphin G, Vokaty S, Ward MP, Wieland B, Lindberg A (2015). One Health surveillance –more than a buzz word?. Prev Vet Med.

[44] Moskalenko L, Schulz K, Nedosekov V, Mõtus K, Viltrop A (2024). Understanding smallholder pigkeepers'awareness and perceptions of African swine fever and its control measures in Ukraine. Pathogens.

[45] Petrik MS, Ivanov OV, Shevchenko VM (2023). Epidemiology of zoonotic diseases in Ukraine:Current status and challenges. Ukrainian Journal of Public Health.

[46] Beauté J, Innocenti F, Aristodimou A, Špačková M, Eves C, Kerbo N, Rimhanen-Finne R, Picardeau M, Faber M, Dougas G, Halldorsdottir AM, Jackson S, Leitēna V, Vergison A, Borg ML, Pijnacker R, Sadkowska-Todys M, Martins JV, Rusu LC, Grilc E, Estévez-Reboredo RM, Niskanen T, Westrell T (2024). Epidemiology of reported cases of leptospirosis in the EU/EEA, 2010 to 2021.. Euro Surveill.

[47] Ivakhiv O, Vyshnevska N, Iosyk I, Vyshnevska Y, Zavidniuk N (2025). A diagnostically challenging leptospirosis case in a serviceman in a combat zone. Mil Med.

[48] Pyskun O, Richter MH (2025). Look and you will find –a literature review of new strains of Leptospira spp.,. 2000–2025. FEMS Microbiol Rev.

[49] Zubach O, Ben I, Zadorozhnyi A (2025). Ukrainian population awareness regarding leptospirosis. Przegl Epidemiol.

[50] Cobby TR, Eisler MC (2024). Risk of rabies reintroduction into the European Union as a result of the Russo-Ukrainian war:A quantitative disease risk analysis. Zoonoses Public Health.

[51] Güner AE, Küçükoğlu MB, Aktura B, Kıran P (2025). Epidemiological and clinical characteristics of West Nile virus infections in Istanbul, Türkiye:A population-based cohort study. BMC Infect Dis.

[52] Kannan A, Chen R, Akhtar Z, Sutton B, Quigley A, Morris MJ, MacIntyre CR (2024). Use of open-source epidemic intelligence for infectious disease outbreaks, Ukraine, 2022. Emerg Infect Dis.

[53] Ministry of Health of Ukraine (2023). Annual report on infectious diseases in Ukraine. https://moz.gov.ua/annual-report-2023.

[54] Salajegheh Tazerji S, Magalhães Duarte P, Gharieb R, Szarpak L, Pruc M, Rahman MT, Rodriguez-Morales AJ, Ilyas MF, Ferreira MNS, Malik YS, Kalantari R, Shahrokhabadi A, Jafari N, Shahabinejad F, Maleki Y, Montajeb S, Mehrpouya R, Ahmadi H, Vazir B, Kabir F, Shehata AA (2025). Migratory wave due to conflicts:Risk of increased infection from zoonotic diseases. Transbound Emerg Dis.

[55] World Health Organization (2023). Global tuberculosis report 2023. https://www.who.int/publications/i/item/9789240083851.

[56] Bajer A, Alsarraf M, Topolnytska M, Tolkacz K, Dwuznik-Szarek D, Rodo A (2023). Vector-borne parasites in dogs from Ukraine translocated to Poland following Russian invasion in 2022. Parasit Vectors.

[57] Rabinowitz PM, Kock R, Kachani M, Kunkel R, Kaplan B, Daszak P (2018). A planetary vision for One Health. BMJ Glob Health.

[58] Rodyna N, Kuzin I, Maiboroda V, Gerilovych A, Pohorielova O, Kupriianova T, Mohylna L (2025). Approaches to epidemiologic surveillance, diagnosis and prevention of West Nile fever in the Kyiv region. One Health Journal.

[59] Zolotukhin O, Tril V, Volkova A, Konechnyi Y (2024). Lyme disease in Ukraine in 2000–2023.. Przegl Epidemiol.

[60] Domen J, Aabenhus R, Balan A, Bongard E, Böhmer F, BralićLang V, Bruno P, Chlabicz S, Colliers A, García-Sangenís A, Ghazaryan H, Kowalczyk A, Jensen S, Lionis C, van der Linde TM, Malania L, Pauer J, Tomacinschii A, Vellinga A, Zastavnyy I, Goossens H, Butler CC, van der Velden AW, Coenen S (2025). The effect of a GP's perception of a patient request for antibiotics on antibiotic prescribing for respiratory tract infections:Secondary analysis of a point-prevalence audit survey in 18 European countries. BJGP Open.

[61] Stepanskyi D, Ishchenko O, McGann P, Lapa Y, Koshova I (2025). Genomic and phenotypic characterization of respiratory pathogens from CF pediatric patients from Dnipro, Ukraine. BMC Pulm Med.

[62] World Health Organization (2025). Ukraine's resilience during war time to strengthen primary health care. https://extranet.who.int/uhcpartnership/story/ukraines-resilience-during-war-time-strengthen-primary-health-care..

[63] Kuzin I, Matskov O, Bondar R, Lapin R, Vovk T, Howard A, Vodianyk A, Skov R, Legare S, Azarskova M, Al-Samarrai T, Barzilay E, Vitek C (2023). Notes from the field:Responding to the wartime spread of antimicrobial-resistant organisms —Ukraine, 2022. MMWR.

[64] Lebreton F, Kondratiuk V, Kovalchuk V, Pfennigwerth N, Luo TL, Jones BT, Fomina N, Fuchs F, Hans JB, Eisfeld J, Ong A, Gatermann S, Bennett JW, McGann P (2025). High genetic relatedness between multidrug-resistant bacteria before and after the 2022 invasion of Ukraine. Genome Med.

[65] McGann P, Luo TL, Martin MJ, Dao HD, Kovalchuk V, Kondratiuk V, Kovalenko I, Plaza BJ, Kettlewell JM, Anderson CP, Smedberg JR, Ong AC, Maybank R, Kwak YI, Hawley-Molloy JS, Lebreton F, Bennett JW (2025). Foreign combatants wounded in Ukraine colonized by extensively drug-resistant organisms:A potential source of global dissemination. J Infect Dis.

[66] Antonenko A, Borysenko A, Melnichuk F, Tkachenko I (2024). Current status of the legal framework in the plant protection and ecology and hygiene monitoring domain in Ukraine. One Health Journal.

[67] Chechet O, Shulyak S, Kobish A, Malimon Z, Omelchun Y (2023). Monitoring of contaminants chemical and biological origin in feed for productive and non-productive animals in 2021 in Ukraine. One Health Journal.

[68] Kolomiiets Y, Butsenko L, Yemets A, Blume Y (2024). The use of PGPB-based bioformulations to control bacterial diseases of vegetable crops in Ukraine. Open Agric J.

[69] Malimon Z, Kochetova H, Gusak L, Shuliak S (2023). Radiation situation in the contaminated territories of Ukraine in the post-Chornobyl period from 2013 to 2022.. One Health Journal.

[70] Prosyanyi S, Horiuk Y, Svintsitska O (2025). The impact of non-ionizing radiation on the quantitative and qualitative indicators of chicken meat products. One Health Journal.

[71] Simiachko O, Mykhailova H (2025). The market of plant protection products in Ukraine. Commodity Sci Technol Eng.

[72] Kornus A, Kornus O, Liannoi Y, Danylchenko O, Lutsenko S (2025). Oncologic burden in Ukraine:Regional inequalities and environmental risk factors. Geospat Health.

[73] Sweileh WM (2022). Global research activity on mathematical modeling of transmission and control of 23 selected infectious disease outbreaks. Glob Health.

[74] Marou V, Vardavas CI, Aslanoglou K, Nikitara K, Plyta Z, Leonardi-Bee J, Atkins K, Condell O, Lamb F, Suk JE (2024). The impact of conflict on infectious disease:A systematic literature review. Confl Health.

[75] Zanella G, Sternberg-Lewerin S, Dürr S (2023). AMR surveillance in livestock systems:Gaps and priorities. Front Vet Sci.

